# Infant manual performance during reaching and grasping for objects moving in depth

**DOI:** 10.3389/fpsyg.2015.01142

**Published:** 2015-08-04

**Authors:** Erik Domellöf, Marianne Barbu-Roth, Louise Rönnqvist, Anne-Yvonne Jacquet, Jacqueline Fagard

**Affiliations:** ^1^Department of Psychology, Umeå University, Umeå, Sweden; ^2^Laboratoire Psychologie de la Perception, Centre National de la Recherche Scientifique-Université Paris Descartes, Paris, France

**Keywords:** infants, reaching, grasping, handedness, moving objects

## Abstract

Few studies have investigated manual performance in infants when reaching and grasping for objects moving in directions other than across the fronto-parallel plane. The present preliminary study explored object-oriented behavioral strategies and side preference in 8- and 10-month-old infants during reaching and grasping for objects approaching in depth from three positions (midline, and 27° diagonally from the left and right). Effects of task constraint by using objects of three different types and two sizes were further examined for behavioral strategies and hand opening prior to grasping. Additionally, assessments of hand preference by a dedicated handedness test were performed. Regardless of object starting position, the 8-month-old infants predominantly displayed right-handed reaches for objects approaching in depth. In contrast, the older infants showed more varied strategies and performed more ipsilateral reaches in correspondence with the side of the approaching object. Conversely, 10-month-old infants were more successful than the younger infants in grasping the objects, independent of object starting position. The findings regarding infant hand use strategies when reaching and grasping for objects moving in depth are similar to those from earlier studies using objects moving along a horizontal path. Still, initiation times of reaching onset were generally long in the present study, indicating that the object motion paths seemingly affected how the infants perceived the intrinsic properties and spatial locations of the objects, possibly with an effect on motor planning. Findings are further discussed in relation to future investigations of infant reaching and grasping for objects approaching in depth.

## Introduction

Whilst the majority of research on infant’s reaching and grasping has been concerned with stationary objects, it has been demonstrated that infants begin reaching for stationary objects and objects moving in the horizontal plane at approximately the same age and with similar precision (von Hofsten and Lindhagen, 1979; von Hofsten, 1980). Consequently, common underlying mechanisms with a common developmental course for both types of reaches have been suggested (Spelke and von Hofsten, 2001). However, it is unclear whether a similar claim can also be made for infant hand preference when reaching for stationary and moving objects. Additionally, whether the motion path and/or object size may affect infant reaching, grasping and hand preference when reaching for moving objects is not fully understood.

The degree of variability in hand preference for reaching and grasping during infancy seems to be influenced by task constraints and complexity such as object size (Fagard and Jacquet, 1996), whether the object to be grasped encourages exploratory behavior (Fagard and Lockman, 2005), and the spatial location of the object (Fagard, 1998). In addition, Wentworth et al. (2000) observed that infants displayed more right-handed reaches for oscillating objects than for static objects, significantly so at 5 and 8 months. With regard to moving objects, infants have been observed utilizing an ipsilateral pursuit strategy for horizontally moving objects at a slow speed, but a contralateral intercept strategy with one preferred hand for faster moving objects (von Hofsten and Lindhagen, 1979; von Hofsten, 1980). For objects moving horizontally along a circular trajectory, infants at 8 months have shown a preferred use of the right hand to reach and grasp regardless of direction, while infants at 6 months generally start to reach with the ipsilateral hand before eventually grasping with the contralateral, and 10-month-olds have displayed various, although largely successful, strategies including frequent bimanual reaching and ipsilateral pursuit (Fagard et al., 2009).

Very few studies have explored infant reaching for objects moving in directions other than across the fronto-parallel plane and, as far as we know, only one including observations of hand use (Wentworth et al., 2000). In that study, it was found that younger infants (5 and 8 months) predominantly reached with the left hand (60%) for objects approaching from beyond reach, whereas older infants (11 months) had a slight predominance for right-handed reaches (57%). Thus, the aim of the present study was to further explore the characterization of infants’ hand use strategies when reaching for objects approaching in depth from far to near. An additional impetus was to pursue the effects of task constraints on the infants’ reaching organization in this particular setting. To this end, the study used three sets of objects of two different sizes moving in depth from three positions toward the infant (starting at far distance position of either the midline, or to the right- or left of the infants, and moving to a near center endpoint).

## Materials and Methods

### Participants

Participants were six healthy 8-month-old (1 girl, 5 boys; mean age 248.5 days, range 240–259 days) and six 10-month-old (4 girls, 2 boys; mean age 311.2 days, range 305–320 days) infants. Two additional infants (one 8-month-old and one 10-month-old, both females) participated in the study, but had to be excluded from further analysis as they did not perform reach-to-grasp movements on any of the trials. All infants were healthy, with no known sensory, motor, or neurological impairments. The study was approved by the local ethics committee of the CNRS-Université Paris Descartes, involved informed parental consent signed before testing and was conducted in accordance with the Declaration of Helsinki.

### Procedure

**FIGURE 1:**
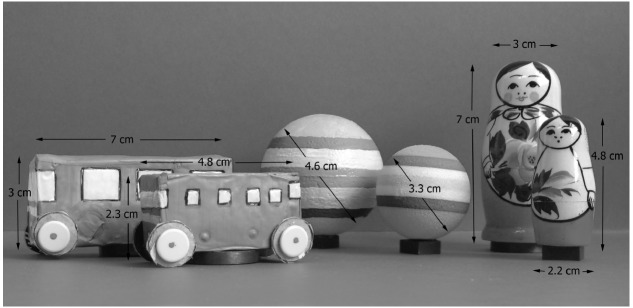
**The experimental objects**.

**FIGURE 2:**
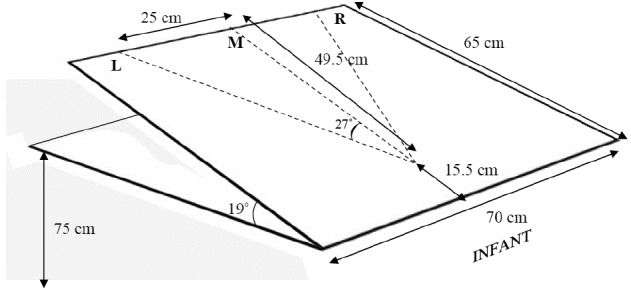
**The experimental set-up**.

The participants were seated in a baby high-chair in front of a testing table which had a 19° angled plywood board (70 × 65 cm) mounted on top. Two infants (both 10 months old, one female and one male) refused to sit in the high-chair but completed the experiment sitting on the lap of their mother at an equivalent height as if sitting in the chair. The reaching targets consisted of three sets of objects (ball, doll, bus) of either a large or a small size (Figure 1). Each of the six objects had a magnet fixated to its base. A second magnet, mounted to a stick, attracted the object magnet from underneath the plywood board and was used to manually transport the object toward the infant. Testing began when the infant was judged to be in an alert state. The objects, initially hidden from the infant, were presented approaching from three positions: midline, and 27° diagonally from the right and left, respectively. The distance between the midline object starting position and the right/left object starting positions was 25 cm in each direction. The object trajectories from the respective starting positions converged at the same endpoint, positioned at a distance of 15 cm in front of the infant (see Figure 2). Based on the mean of 85 trials, object velocity was 5.2 cm/s (SD 0.9) regardless of type of object or starting position.

The experimental set-up is depicted in Figure 2. Each object was presented once in each starting position (18 trials). For all infants, objects were first presented approaching from the midline in a randomized order and then from either the right or left position, with both side and objects following a randomized and counterbalanced order with regard to number of presentations per position. Two video cameras (Canon MVX 460) supplied recordings of each trial from two different angles (in front and above).

To enable a comparison of hand preference between the age groups, the infants were tested with the baby handedness test (BbHtest; Sacco et al., 2006) which consists of five items to test simple grasping and two items to test precision grasping. The objects used to test simple grasping were small baby toys: three Playmobil figurines, one musical toy (maracas), and a teether. For precision grasping, one of the tasks consisted of removing a very thin red tube (diameter 6 mm) from a slightly shorter transparent tube into which it was inserted with only the top protruding, and the other task consisted of grasping a small toy horse inserted in a container (height 30 mm).

### Data Extraction and Analysis

Of the 216 trials obtained from the participating infants, 18 had to be excluded due to the infant failing to perform reaching movements, inattentiveness (looking away from the object), technical difficulties (object falling off the attractor magnet), or fussiness. Of the remaining 198 trials, 12 reaches led to no attempt to grasp and 186 led to unimanual grasping or attempts to grasp. For all infants, one hand could always be identified touching the object before the other and, thus, reaching and grasping were consistently considered as unimanual movements.

For each reaching trial, reaching onset, hand use, object start, object touch, and, if applicable, object grasp, and hand position at grasp, were identified by frame-by-frame analyses (25 frames/s). Object start was determined as the first frame when the object moved toward the infant. Reaching onset was defined as the frame when the hand was judged to start moving toward the object while the infant at the same time was visually fixating and following the object. If there was a grasping attempt leading to a successful grasp, the frame when the object was lifted was treated as time for object grasp. The following parameters were derived: initiation time (IT, time difference between object start and reaching onset), reaching duration (RD, time from reaching onset to object touch), grasping duration (GD, time from object touch to object grasp). Further, a still image of every first frame before object touch for the reaches that resulted in a grasp was procured from the video recordings and used for scoring quality of hand opening in relation to the object. Following Fagard (2000), we coded either of three possible outcomes: “hand not open” (score 0), “part of the object within hand” (score 1), “whole object within hand” (score 2).

Inter-judge reliabilities between two judges (ED, AYJ) who independently scored the trials for 2 infants were 100% for hand use, 97% for object start (mean difference 1.1 frames), 89% for reaching onset (mean difference 2.1 frames), 100% for object touch (mean difference 1.2 frames), and 92.4% for object grasp (mean difference 1.5 frames). Scoring of quality of hand opening prior to object touch was based on a consensus between two judges evaluated for each trial (ED, JF).

For the BbHtest, a laterality index (LI) was calculated as follows: (Number of right hand grasps-Number of left hand grasps)/(Total number of grasps). According to the results of a previous study on the distribution of handedness (Fagard et al., 2015), we categorized the infants as mainly right-handers (LI > +0.30), mainly left-handers (LI < –0.30) and mixed-handed (-0.30 ≥ LI ≤ + 0.30). Overall, 7 infants were found to be mainly right-handers, two to be mainly left-handers (one left-handed in each age group), and three infants were judged as being mixed-handed. Since we found no significant difference between the two groups for LIs and for the relative percentages of right-handers and mixed-handed children, we do not present further results of the handedness test for static objects.

### Statistical Analysis

Separate ANOVAs were used to analyze the effects of age (2), object starting position (3), type of object (3), and object size (2) on all outcome measures. All analyses are reported in the Results section when they occurred. *Post hoc* comparisons were made with the LSD test. The pre-set alpha level was 0.05 but, due to the small sample size, *post hoc* testing was employed also when main effects were close to significant.

## Results

### Hand Preference for Reaching

Of the total 198 reaching movements, 115 were made with the right hand (58.1%) and 83 with the left hand (41.9%). Thus, the majority of reaching was right-handed, more so in 8- than in 10-month-olds, and less so when the object started approaching from the left position (see Table 1). ANOVA on the amount of right-hand reaching as a function of age and object starting position showed no significant main effect for age. The effect of object starting position was close to significant (*p* = 0.075). *Post hoc* testing showed that starting position had no significant effect at 8 months but the difference in percentage of right-hand reaching between the right and left object starting positions was close to significant at 10 months (*p* = 0.06). Object size and type of object had no significant effect on the hand used for reaching.

**TABLE 1:** **Percent (%) of right-handed reaches as a function of age and object starting position**.

	**8 months %**			**10 months %**		
**% RH Reach**	**L**	**M**	**R**	**L**	**M**	**R**
RH	58.3	75	71.7	33.1	55.5	58.3

RH, right hand; L, left; M, midline; R, right.

### Hand Use for Successful Grasping

**FIGURE 3:**
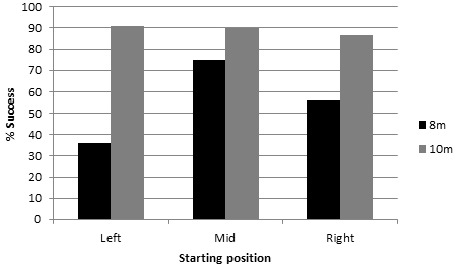
**Frequency of successful grasping in the three object positions as a function of age group**.

Of the 198 reaching movements, 186 were followed by an attempt at grasping the object. Of these 186 attempts, 134 were successful (72%) and 52 led to failure. A first analysis of mean success as a function of age, type of object, object size and starting position indicated that object type and size had no effect on success, and with no significant interaction between them or with the other variables. Thus, ANOVA was performed on the amount of success as a function of age (8 months, 10 months) and object starting position (midline, right, left, repeated measures) after collapsing object types and sizes. Grasping success was found to be significantly more frequent at 10 months (90.9%) than at 8 months (61.9%), [*F*(1,18) = 5.1, *p* = 0.049]. The effect of position did not reach significance (*p* = 0.12) although a *post hoc* test showed that grasping success was significantly higher when the objects were presented at midline than to the left (*p* = 0.029). No significant interaction between age and position was found (*p* = 0.09). However, *post hoc* testing revealed that for the 8-month-olds, grasping success was higher when the object starting position was at midline (75%) than at the left starting position (*p* = 0.004), whereas for the 10-month-old infants, the rate of success was very similar for the three starting positions (90, 86.7, and 91% at the middle, right, and left positions, respectively; see Figure 3).

Of the 134 successful grasping performances, 71 were made by the right hand (mean number of success RH: 57.5%) and 63 by the left hand (mean number of success LH: 42.5%). In general, infants were more likely to grasp objects starting from the midline with the right hand (56%) and objects starting from the left with the left hand (LH: 56%). However, an ANOVA performed on the percentage of right hand use for grasping as a function of starting position and age showed no main effect for position (*p* = 0.26), no main effect for age (*p* = 0.24), and no interaction effect (*p* = 0.70).

Mean success of all trials where the infant tried to grasp the object (success and failure) was 72.7% for the right hand, 78.2% for the left hand, a non-significant difference. Thus, success at a given position was not a function of the hand used for grasping.

### Temporal Parameters

Repeated measures ANOVA performed for the temporal parameters showed no significant main effects for object type or size, and no significant interactions. Further, having collapsed objects and sizes, no significant main effects for age or position and no significant interaction were found. Still, the infants at 10 months generally displayed slightly increased temporal parameters than infants at 8 months (Table 2). IT tended to be longer for the left object starting position (*M* = 7.0 s) than for the right starting position (*M* = 6.08 s), and for the left hand (*M* = 6.8 s) compared with the right hand (*M* = 6.3 s). However, none of these differences were significant. No significant hand use differences were found for RD or GD.

**TABLE 2:** **Means and standard deviations for reaching initiation time (IT), reaching duration (RD), and grasping duration (GD) in the three object positions**.

****	**8 months Mean (SD)**			**10 months Mean (SD)**		
**Parameter (s)**	**L**	**M**	**R**	**L**	**M**	**R**
IT	6.6 (1.5)	6.6 (2.9)	5.7 (0.8)	7.3 (2.0)	6.5 (1.4)	6.5 (2.7)
RD	1.4 (0.7)	1.5 (0.7)	1.3 (0.6)	1.3 (0.4)	1.6 (0.8)	1.6 (0.5)
GD	1.3 (0.9)	2.1 (2.4)	1.7 (1.1)	1.7 (1.6)	2.1 (1.8)	1.5 (0.6)
						
**Total**	**L**	**M**	**R**			
IT	7.0 (1.7)	6.6 (2.2)	6.1 (2.0)			
RD	1.3 (0.5)	1.5 (0.7)	1.4 (0.5)			
GD	1.5 (1.3)	2.1 (2.0)	1.6 (0.8)			

SD, standard deviation; s, seconds; L, left; M; midline; R, right; IT, initiation time; RD, reaching duration; GD, grasping duration.

### Hand Opening in Relation to the Object

Repeated measures ANOVA on the hand opening quality score relative to age, object type and object size revealed no main effect of age, but a main effect of object type, *F*(2,18) = 14.2, *p* = 0.0002. The *post hoc* test showed that the quality score was higher (object more within hand) for the doll (mean score = 2.23) than for the bus (mean score = 1.65; *p* = 0.002), and for the ball (mean score = 1.96) than for the bus, *p* = 0.021. There was a significant interaction between age and object, *F*(2,18) = 4.5, *p* = 0.026. *Post hoc* testing showed that the 8-month-olds displayed significantly higher hand opening scores for the ball and the doll than for the bus, whereas the 10-month-olds displayed significantly higher scores for the doll than for the ball and the bus. There was no main effect of object size and none of the other interactions were significant. In general, the right hand tended to generate higher hand opening quality scores (*M* = 1.89, SD = 0.6) than the left hand (*M* = 1.79, SD = 0.53) but this difference was not significant.

## Discussion

The primary aim of the study was to investigate infants’ hand use and reaching and grasping strategies for objects approaching in depth. For each motion condition, the object started either from a midline, right or left position at the upper part of a slanted board. Overall, the majority of the reaching movements made by the participating infants were made by the right arm/hand, although the relative proportion of right- versus left movements was found to be depending on age and object starting position. At 8 months, infants were found more likely to use right-handed reaching at all three starting positions. In contrast, the 10 month-old infants performed significantly more reaching movements with the right arm/hand when the object started from the right than when it started from the left, where there was a majority of reaches made by the left arm/hand.

The fact that 8-month-olds reach predominantly with the right hand is in contradiction to the one previous study made observing infants’ hand use when reaching for approaching objects in the same plane (Wentworth et al., 2000). However, in keeping with Wentworth et al. (2000), we observed that the older infants displayed more varied reaching strategies than the younger ones, and in particular that they often reached with the hand ipsilateral to the side of the object’s starting position rather than primarily using the right hand. This finding, as well as the observation that 8-month-olds more systematically used their right hand rather than being influenced by the spatial constraints of the task, is in keeping with Fagard et al. (2009) regarding 8- and 10-month-old infants reaching for horizontally moving objects. In that study, 8-month-old infants most often reached with their right hand whereas the 10-month-olds changed with the object’s starting position, frequently reaching with the ipsilateral hand. The findings of the present study confirm that the younger infants seem to choose their hand on the basis of hand preference, whereas the older infants better anticipate the hand best suited as a function of the spatial constraint. Anecdotally, the 8-month-old left-hander never used the right hand during the reaching task, whereas the 10-month-old left-hander used the right hand to reach for the right-presented objects at 50% of the presentations (but 0% of the middle presentations).

The finding that the 8-month-old infants chose their preferred hand whatever the spatial condition, whereas the 10-month-old infants were more influenced by the spatial condition is interesting to discuss in relation to the IT outcome. Compared with the younger infants, the time elapsed between the start of the object and reaching onset tended to be longer for infants at 10 months. In keeping with Rosander and von Hofsten (2011), one possible explanation for this finding is that 10-month-old infants have a more developed action plan when observing actions, and thus, make a better estimation (perceptual judgement) of the objects’ motion information (approach speed and trajectory) and, thus, are able to perform better estimations of the object time-to-arrival and its reachability. It could also be associated with what Schaffer et al. (1972) refer to as the “capacity for not-approach,” suggested as being absent before 9 months of age (Schaffer et al., 1972, p. 175) and likely appearing in synchrony with developing frontal lobe functions in terms of, e.g., impulse control (Diamond and Doar, 1989). That the 10-month-old infants seemingly planned/adjusted their reaching onset according to the grasping zone before acting is indicative of a more intention-based, goal-directed focus. Accordingly, the 10-month-old infants may have grasped the objects with a more pronounced intention to explore it, which may affect preferred hand use in infants (Fagard and Marks, 2000).

Infants grasped the objects successfully in 72% of the trials. The high rate of failure, unusual at the investigated ages, was probably due to the fact that the object was presented on a slanted surface so that it sometimes fell when the grasp was not properly prepared. The percentage of success was significantly higher in the 10- than in the 8-month-old infants. This age-related increase in skill for grasping moving objects fits with observations of developmental changes in grasping static objects (von Hofsten and Rönnqvist, 1988; Fagard, 1998). Further, success at grasping was also less frequent in the left starting position condition than in the midline one at 8 months. IT was also slightly longer at the left starting position than at the right position. It could thus be, for instance, that to succeed at grasping the object from the left starting position, infants must take the time to inhibit the spontaneous use of the preferred right hand.

Changes in manual strategies in infant prehension of horizontally moving objects during the first year of life have been observed following a non-linear trend (van Hof et al., 2005; Fagard et al., 2009). The present study supports this suggestion of non-linearity, further adding reaching and grasping for objects approaching in depth to the phenomenon. Developmental studies have established that very young infants prefer ipsilateral hand use when reaching for corresponding ipsilateral stationary objects, then change to mainly using one preferred hand regardless of object position, and later to more flexible strategies (Fagard, 1998; Rönnqvist and Domellöf, 2006; Fagard et al., 2009). Some of these studies used moving objects in horizontal planes, while others used stationary objects. Some differences between the results may stem from the fact that moving objects, to a greater extent than stationary objects, induce changing interactions between intrinsic and extrinsic constraints that are differently expressed in infants’ prehension behavior depending on age, as suggested by van Hof et al. (2005). In addition, differences in demand and outcomes between reaching for horizontally and vertically moving objects could be associated with differences in the respective affordances of vertical and horizontal eye- and head tracking in infants. Studies devoted to eye- and head tracking ability of moving objects in infants at 6- to 12 months have established a developmental difference in terms of less mature vertical than horizontal tracking in young infants (Gredebäck et al., 2005; Jonsson et al., 2009). Assuming that such directional differences in eye- and head tracking ability play a role in infant reaching and grasping moving objects, it is possible that the mainly vertical tracking of approaching objects in the present study was more readily achieved by the older infants (i.e., more mature vertical tracking ability), adding to the task being less complicated for the infants at 10 months compared with those at 8 months.

It should be noted that there are a number of restrictions to the present study that warrant caution when interpreting the results. Thus, the study is best regarded as a first preliminary investigation to aid refinement and further methodological development in future studies. The number of participants is limited, affecting group sizes and inevitably reducing statistical power. Apart from a larger sample, an additional age group consisting of 6-month-old infants would have been desirable with regard to the argumentation concerning non-linearity in infant manual strategies. Optimally, a longitudinal approach, following the same infants from 6 to 12 months, preferably comparing reaching and grasping for both horizontally and vertically presented objects, should be considered. Whilst efforts to use detailed measures were made, the methodology could have been improved by more specific and reliable techniques. Kinematic registration would have enabled subtle differences in lateralized spatio-temporal arm movement organization between the age groups to be more clearly revealed (c.f., Rönnqvist and Domellöf, 2006). This type of technique would also enable measurements of postural displacements during reaching that might have affected the outcome. For example, the propensity toward shorter IT found in the 8-month-old infants in the present study may have been due to more body engagement (tilting the upper-body forward at reaching onset in relation to the in-depth approaching object). This reasoning is in line with investigations of younger infants reaching for stationary objects presented at various distances and beyond the reach of the infant, suggesting that perceived reachability is calibrated in relation to the degree of postural control achieved by the infant (Rochat et al., 1999). Finally, involving measurement of eye-tracking would also be a relevant addition to investigate aspects of side-specific action planning and to control for visual cues.

In conclusion, the present study reports patterns of hand use strategies and preferences in infants at 8- and 10-months-old similar to those obtained in previous studies of infant reaching and grasping for horizontally moving objects. This supports the notion of a related underlying mechanism for infant hand use when reaching for moving objects in both the fronto-parallel and vertical plane. However, to be noted is the prolonged GDs in the present study compared with previous findings related to infant prehension of stationary and horizontally moving objects (e.g., von Hofsten, 1983; von Hofsten and Rönnqvist, 1988). This dissociation indicates that prehension of objects approaching in depth, even slowly moving objects as in this study, is more challenging for the infants in terms of judgements of object speed, distance and size, and perhaps own body sway. In this study, the back to front motion path seemed to affect grasping movement in particular, as expressed by longer GDs, with more hand corrections depending on the object and, consequently, less adjusted hand opening positions prior to object contact and grasp. This would also be in keeping with the suggestion of independent visually guided reaching and grasping movements, with different neural underpinnings and evolutionary origins, and with visual guidance of reaching movements developing prior to visual guidance of grasping during infancy (Karl and Whishaw, 2013). Future studies involving more sophisticated measurement techniques and larger samples of infants will hopefully confirm the preliminary findings reported here, and bring further clarity to the additional questions that they have evoked.

## Author Contributions

ED contributed in conceptualizing and designing the study, was in charge of the data collection, participated in the data analyses and interpreted them, prepared the first draft of the paper, and approved the final draft as submitted. MBR contributed in conceptualizing and designing the study, reviewed and revised the manuscript, and approved the final draft as submitted. LR contributed in conceptualizing and designing the study, co-wrote, reviewed and revised the manuscript, and approved the final draft as submitted. AYJ took part in the data collection and approved the final draft as submitted. JF contributed in conceptualizing and designing the study, took part in the data collection, carried out the data analyses and interpreted them, co-wrote, reviewed, and revised the manuscript, and approved the final draft as submitted.

### Conflict of Interest Statement

The authors declare that the research was conducted in the absence of any commercial or financial relationships that could be construed as a potential conflict of interest.
